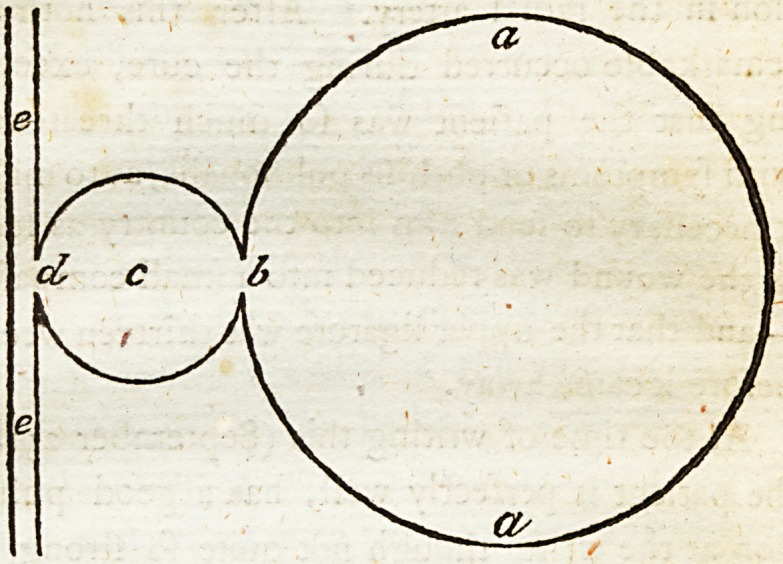# A Case of Varicose Aneurism

**Published:** 1793

**Authors:** H. Park

**Affiliations:** Surgeon to the Liverpool Infirmary.


VIII. *A Caſe of Varicoſe Aneuriſm. By Mr.* H. Park, *Surgeon to the Liverpool Infirmary.*

JOHN HARTLEY, a youth about twenty years of age, preſented himſelf at the Liverpool Infirmary in May, 1791. His account of himſelf was, that he had been bled in the right arm in the preceding January; that he had more than uſual pain at the time, and thought he had been pricked too deep; that he continued his work (that of a wheelwright) for a week after the operation, and then perceived a ſmall hard tumor immediately under the orifice, which had had gradually increafed to its prefent fize, which was fomewhat larger than a walnut. It was then as foft as aneurifmal tumors generally are; had an evident pulfation, and on preffure, or on holding up the arm, difappeared in a confiderable degree, but not entirely; from which it was evident, that the whole of the blood contained in the tumor was not in a ftate of fluidity, but that fome coagulum was formed. The peculiar thrilling fenfation, fo accurately defcribed by Doctors Hunter and Cleghorn, was very plainly perceptible more than half way up to the axilla, and the bafilic vein was a good deal diftended, but not in the degree which they defcribe in their cafes* of varicofe aneurifm, the tumor being ftill on the increafe, though flowly. On the whole, though there was no room to doubt that fome part of the blood that was thrown out from the trunk of the artery was received, and did return, by the trunk of the vein, yet there was reafon to fear that this did not take place in a fufficient degree to fecure the patient from the neceffity of undergoing an operation at no very diftant period; and therefore a guarded opinion was given him, with

* See Medical Obf. and Inq. Vol. II. and III.

directions to refrain from any laborious employment, and to let us see him again if any material change should take place. He called again at the Infirmary a few months afterwards, when it was evident that the tumor was still increasing faster than the distention of the vein.

On the 21st of January following he came into the Infirmary in considerable pain, from a high inflammation upon the arm, with evident marks of suppuration having taken place, and of the tumor being on the point of bursting; this he attributed to accidental cold; but it was probably owing to the sudden increase of the tumor, which had grown rather rapidly during the last month, and was now larger than an ordinary man's hand. An emollient poultice was applied, and a tourniquet was put loosely round the arm, with directions that he should be closely watched day and night. The next morning the skin opened, a small quantity of pus was discharged, the tumor subsided a little, and the patient became considerably easier.

On the 24th in the evening the blood burst forth with considerable force, the tourniquet was tightened, and the Surgeons were called as soon as possible; some time, however, was unavoidably lost. On opening the sac, and removing the coagulum, of which there was a considerable quantity, an orifice was discovered at the bottom of the cavity, not larger than is usually made in venesection, from which arterial blood flowed on loosening the tourniquet. A probe introduced into this orifice sunk nearly an inch deep, but would not pass much more than half an inch upwards or downwards, therefore we did not choose to venture a ligature till we were more sure of the artery; believing there must be an inner cyst. This orifice was cautiously enlarged with the scissars, and was found to lead into a deeper cavity, large enough to contain a moderate sized nutmeg; in this there was not any coagulum, but at the bottom of it was discovered another orifice, of about the same size as the former; and a probe, introduced into this, passed readily upwards and downwards in the cavity of the artery. Directed by this probe, a ligature was passed underneath the artery, above the orifice, and tied. On loosening the tourniquet the blood flowed out as freely as before; another ligature was passed in the same manner below the orifice, and tied; and on loosening the tourniquet now, no farther hæmorrhage appeared.

I ſhould have mentioned that early in the operation an external branch of the artery bled freely, and was tied.

A ſection of theſe cyſts would exhibit ſome ſuch appearance as this :

*a a*, the firſt or outer cyſt, *b*, the orifice leading into the ſecond or deeper cyſt, *c*, at the bottom of which was ſeen the orifice *d*, leading into the cavity of the artery marked *e e*.

We kept the wound open more than half an hour, (waiting to ſee if any more hæmorrhage would take place) and then it was cloſed up as eaſily as poſſible, by merely bringing the edges together by means of long ſlips of adheſive plaſter.

The next day the œdematous ſwelling that had taken place in conſequence of the long continuance of the tourniquet, in the ſpace of time between the burſting out of the blood and the concluſion of the operation, was conſiderably diminiſhed; there was comfortable warmth and feeling in the fore arm, and a faint pulſation in the radial artery. After this nothing remarkable occurred during the cure, excepting that the patient was ſo much threatened with ſymptoms of phthiſis pulmonalis, as to make it neceſſary to ſend him into the country as ſoon as the wound was reduced into a ſmall compaſs; —and that the upper ligature was thirteen weeks before it came away.

At the time of writing this (September 29th) the patient is perfectly well, has a good pulſation at the wriſt, though not quite ſo ſtrong as in the other, but the arm is equally ſtrong and muſcular, and has perfect motion; and he follows the trade of a ſhip carpenter, to which he has lately bound himſelf.

It will probably be aſked, if this was really in any degree a caſe of varicoſe aneuriſm, where was the vein? and, if it was ſituated between the outer and inner cyſts, why was it not diſcovered? and poſſibly I might be critic enough to aſk theſe queſtions myſelf, had this operation been performed by any other perſon; but I

muſt muſt beg leave to ſubmit the following conſiderations to experienced and unprejudiced practitioners. Firſt, the inflammation and ſuppuration had contributed to occaſion ſome degree of confuſion of parts. Secondly, the outer cyſt was ſo thick as to prevent any veſſels from being viſible that might run underneath it. Thirdly, the two ſides of the vein, when it was empty, might very readily be ſo cloſely preſſed together as to admit of a probe paſſing through its very center into the inner cyſt without the cavity of the vein being ever diſcovered; and if ſuch was the ſituation of the vein, it is, perhaps, happy for the patient it was not diſcovered; as there might have been ſome danger of its miſleading us to tie it inſtead of the artery. I muſt beg it may be farther conſidered, that the patient had been diſtreſſed by having the tourniquet unavoidably kept on the arm a conſiderable time before the operation could be begun; that it was obliged to be performed by candle light, and proved very tedious and perplexing; and that the patient was very much exhauſted: under which circumſtances, I conceive, I ſhall ſtand perfectly excuſable in attending only to the main object of my purſuit, viz. to diſcover and ſecure the wound in the artery, and ſo terminate my patient's ſufferings with as much expedition as could be conſiſtent with perfect ſafety

As Dr. Hunter judiciouſly and humanely publiſhed his caſes with a view to prevent patients being unneceſſarily expoſed to a ſevere operation, ſo I think it incumbent on me to ſtate the above, as a caution to inexperienced practitioners, whenever they meet with ſuch appearances as the Doctor has deſcribed, but in a leſs degree, to be well aſſured that the veins will really dilate ſufficiently to take off the whole of the blood poured out by the artery, before they give ſuch a prognoſtic as may lull the patient into a deluſive and dangerous ſecurity.

## Figures and Tables

**Figure f1:**